# General Public Awareness Toward Vitamin D Deficiency in Qassim, Saudi Arabia

**DOI:** 10.7759/cureus.63967

**Published:** 2024-07-06

**Authors:** Muhammad A Almutairi, Omer AlYahia

**Affiliations:** 1 Family Medicine, Family Medicine Academy, Buraidah, SAU; 2 Family Medicine, Qassim Health Cluster, Buraidah, SAU

**Keywords:** saudi arabia, sun exposure, knowledge, awareness, vitamin d deficiency

## Abstract

Introduction

Vitamin D, essential for various bodily functions, exists as D2 and D3, synthesized from plant and animal sources. Deficiency, linked to reduced intake, sun exposure, or metabolic disorders, poses health risks like diabetes and cardiovascular diseases. Management includes fortification, supplements, and individual assessment to avoid toxicity. Our study aimed to assess the public awareness of vitamin D deficiency in Qassim, Saudi Arabia, examining its relationship with sociodemographic factors.

Methodology

This cross-sectional study was conducted in the Qassim Region, Saudi Arabia, employing convenient non-probability sampling among a population of 375 individuals. Data collection was facilitated through a validated questionnaire, and subsequent coding, data entry, and analysis were executed using IBM SPSS Statistics for Windows, Version 27.0 (Released 2020; IBM Corp., Armonk, NY, USA).

Results

Our study included 375 adults in Qassim, Saudi Arabia, revealing varying vitamin D deficiency awareness levels. Most were women (n = 204, 54.4%) residing in urban areas (n = 293, 78.1%). At the same time, 77.6% (n = 291) had prior knowledge of vitamin D and fewer practiced habits conducive to its synthesis, with 47.7% (n = 179) including vitamin D-rich foods and 39.2% (n = 147) exposing themselves to sunlight. Family/friends (n = 99, 26.4%) were the primary information source. Participants scored (n = 183, 48.8%) good or high on vitamin D awareness. Education level (p = 0.028), urban residence (p = 0.001), employment status (p = 0.032), and income (p = 0.001) significantly influenced awareness, and gender, age, and marital status showed no significant associations with vitamin D awareness.

Conclusion

Our study shows that the majority have prior knowledge of vitamin D, yet significant gaps exist in awareness of vitamin D deficiency among adults in Qassim, Saudi Arabia. Education, urban residence, employment, and income were critical determinants of awareness, underscoring the need for targeted educational interventions. There is a need to provide information about vitamin D and its other aspects through various media, such as television and social platforms.

## Introduction

Vitamin D is the name used to describe a group of molecules that play essential roles in the human body and have been described as an important nutrient. These molecules are fat-soluble steroid compounds with similar chemical structures and biological functions but different origins [1]. Vitamin D may occur in multiple forms, but two of these forms are majorly found in the human body, namely vitamin D2 (or ergocalciferol) and vitamin D3 (or cholecalciferol). Historically, vitamin D1 was the name used to describe a substance that was isolated in the 1920s, but it was later found to be a mixture of vitamin D2 and tachysterol [2]. Vitamin D2 is a plant-derived nutrient that is produced in yeast by the irradiation of ergosterol. On the other hand, vitamin D3 is an animal-derived compound, as it is made in the skin cells when exposed to UV-B rays from the sun [1,2].

Both vitamins D2 and D3 are converted into their respective hydroxylated forms (i.e., 25-hydroxy vitamin D2 (25-OH-D2) and 25-hydroxy vitamin D3 (25-OH-D3), respectively) by the catalytic activity of a liver enzyme known as 25-hydroxylase. However, the active form of vitamin D is 1,25-dihydroxy vitamin D, which is obtained by another hydroxylation of both 25-OH-D2 and 25-OH-D3 through an enzyme in the kidney known as 1-alpha-hydroxylase [3]. The levels of 25-hydroxy vitamin D are measured in blood or serum to identify if the body has a sufficient amount of vitamin D to perform its functions. However, the required threshold to define vitamin D deficiency is still variable. Most conservative definitions state that serum 25-hydroxy vitamin D levels should be less than 25 nmol/L to declare deficiency [4], while the International Society for Clinical Densitometry and the International Osteoporosis Foundation recommend that older individuals should have a minimum of 30 ng/mL serum 25-hydroxy vitamin D to avoid fractures in cases of falls [3,5]. Recently, a serum 25(OH)D level of less than 20 ng/mL or 50 nmol/L has been categorized as vitamin D deficiency, and it was found to be associated with an increased risk of skeletal issues such as bone fractures. Besides, the serum 25(OH)D level reaching below 12 ng/mL or 30 nmol/L was categorized as severe vitamin D deficiency [6].

Vitamin D has been identified to perform multiple important functions in the human body. The most predominant role of vitamin D is in the process of calcium and phosphorus absorption from the intestine. The active form of vitamin D increases the resorption of these nutrients by bones and reduces their excretion from the kidneys [3]. Besides the functions of vitamin D in skeletal system maintenance, vitamin D also modulates mechanisms involved in the processes of growth, inflammation, immunity, and gene expression [1,7,8]. Due to the multisystemic roles of vitamin D, the deficiency of this vitamin can be a risk factor for a variety of health complications, such as diabetes, musculoskeletal disorders, cardiovascular diseases, dementia, autism, cancer, polycystic ovarian syndrome, and male hypogonadism [9,10].

Vitamin D deficiency can be caused by multiple factors. The most prevalent factor contributing to vitamin D deficiency is a reduction in the intake of diet-based vitamin D. Moreover, certain disorders that prevent the absorption or disrupt the metabolism of vitamin D may also cause vitamin D deficiency. Reduced skin exposure to sunlight is also an essential factor leading to vitamin D deficiency in the body. Covering skin with clothing or sunscreen during sun exposure also inhibits vitamin D synthesis. Moreover, vitamin D deficiency can also be caused by errors in the mechanisms that synthesize active forms of vitamin D in the body. For instance, patients affected by liver diseases (such as cirrhosis) may have defective enzymes that cannot convert vitamins D2 and D3 into their respective hydroxylated forms. Similarly, patients with kidney disorders (such as renal failure) may also develop vitamin D deficiency due to diminished activity of the enzyme 1-alpha-hydroxylase to produce the active form of vitamin D, i.e., 1,25-dihydroxy vitamin D [3].

Preventive and therapeutic measures are recommended for managing vitamin D deficiency. General food fortification programs may improve the level of vitamin D in a given population. People who cannot have sufficient sun exposure (20 minutes daily) should be advised to take vitamin D supplements. Supplements for vitamin D are available in different forms and compositions. The most effective outcomes for the revival of required vitamin D levels in the body have been reported by using vitamin D preparations composed of cholecalciferol or vitamin D3. However, it is necessary to evaluate the individual level of deficiency for vitamin D, as well as the underlying causes and risk factors, before prescribing the amount of vitamin D supplementation to avoid toxicity [3,6].

Day et al. presented the findings of a study carried out in the UK to identify the awareness and behaviors of parents of young children (aged 0-2 years old) regarding vitamin D deficiency. The study recruited quantitative and qualitative methods and collected data from 194 parents through online surveys. Focus group participation and interview-based collected information were analyzed via thematic analysis. It was noted that the majority (80%) of the parents wanted to know more about vitamin D requirements in their children. Overall, parents were not well aware of the subject and were not satisfied with the information provided for vitamin D deficiency [11]. Clark et al. also reported a general lack of knowledge about vitamin D deficiency and misconceptions about food fortification programs among different ethnic populations in the UK. These findings were observed from the outcomes of two pilot studies conducted on the topic. One study was carried out by collecting information from two groups, i.e., people at risk of vitamin D deficiency (Caucasian adults older than 65 years and non-Caucasians, i.e., Black, South Asians, Far Eastern, and Middle Eastern) and people at reduced risk of vitamin D deficiency, i.e., British Caucasians not avoiding the sun exposure. The second study recruited data from South Asian females [12].

In Canada, Boland et al. conducted a survey to analyze university students’ awareness of vitamin D deficiency. Data from more than 1,000 students was collected through questionnaires. The analysis revealed an overall poor awareness level among the studied subjects, as only 8% of the study participants could provide correct answers about the recommended intake of vitamin D. Only 14% of the students knew about the required sun exposure time to synthesize sufficient vitamin D in the body [13].

Zhou et al. also conducted a cross-sectional study in China to identify the knowledge and attitudes of university students regarding vitamin D deficiency. The study enrolled more than 500 medical students and collected data through questionnaires. Inadequate knowledge and poor behaviors were noted when the data was analyzed, as only 5.6% of the students reported using vitamin D supplements, while low consumption of vitamin D-rich foods was also observed in the studied population [14].

Toher et al. also reported that the majority (71%) of pregnant women residing in Ireland were unaware of vitamin D deficiency. The researchers conducted a cross-sectional study and analyzed data collected from over 100 pregnant women from different origins, including Asia, sub-Saharan Africa, North Africa, the Middle East and North Africa (MENA), and Ireland. The majority of the women from the MENA region (88%) were found to be at risk of vitamin D deficiency [15].

Ibrahim et al. published the findings of a cross-sectional study in the UAE to identify the awareness level of vitamin D deficiency in the local population. This study was conducted by recruiting data from more than 400 participants through surveys. It was observed that the prevalence of vitamin D deficiency was higher in females (83%), compared to males (42%). This might be due to excessive use of sunscreen, as reported by 55% of the female participants in this study. Also, knowledge about sunlight exposure being the main source of vitamin D synthesis was reported in only 21.4% of the study participants [16]. Likewise, Salmanpour et al. reported the results of a cross-sectional study to assess vitamin D deficiency awareness among people living in Sharjah, UAE. This study analyzed the data collected from more than 500 participants recruited through convenience sampling and questionnaires. The study outcomes revealed that 97% of the participants never tested their vitamin D levels. In comparison, the majority (77%) tend to avoid sunlight exposure, and only 43% of the study participants knew sunlight was a source of vitamin D synthesis [17].

Vitamin D deficiency prevalence in Saudi Arabia is very high, ranging from 40% to 80% of the adult population [18]. AlGarni et al. reported the findings of a cross-sectional study carried out to assess the general public awareness of the Saudi population residing in Taif City toward vitamin D deficiency. Among 431 study participants, only 23.9% scored better when tested for awareness about the subject. Moreover, 46.6% of the study participants reported being affected by vitamin D deficiency [18]. Alkalash et al. published the findings of an analytical cross-sectional study to analyze vitamin D deficiency awareness among the people living in the Al Qunfudhah governorate of Saudi Arabia. This study recruited data from more than 460 participants through questionnaires. The study identified poor knowledge and attitudes toward vitamin D deficiency among the study participants, as only 17.4% of participants knew sunlight exposure was a source of vitamin D synthesis [19]. Alamoudi et al. assessed the general public awareness about vitamin D deficiency in the local population residing in Jeddah, Saudi Arabia. For this purpose, the authors conducted a cross-sectional study by collecting data from more than 1,000 participants through questionnaires. Data analysis revealed that the mean level of knowledge among the studied population was 39.3%, and 69.4% of the participants were aware of the role of vitamin D in immunity. In comparison, 86.2% were aware of the role of sunlight in the synthesis of vitamin D in the body [20].

Given that there is a high prevalence of vitamin D deficiency in Saudi Arabia and that the same has effects on the well-being and quality of life of individuals and costs the healthcare system, it is necessary to increase population awareness about the problem. In order to increase population awareness, the first step is to assess their knowledge levels and areas of deficiency to devise target awareness strategies. This research will provide important insights into the issue and help develop interventions for increasing population awareness about vitamin D deficiency and reducing the burden of vitamin deficiency in the population.

Objectives

The study aimed to assess the general public awareness of vitamin D deficiency in Qassim, Saudi Arabia. Additionally, we aimed to identify the relationship between the level of awareness and different sociodemographic factors.

## Materials and methods

Study design

A cross-sectional study was conducted among the general population of Qassim, aged 18 years and above.

Study area

This study was conducted in Qassim, Saudi Arabia, from April 2023 to April 2024.

Study population

The adult general public population was recruited as the study population. The eligibility criteria are mentioned in Table 1.

**Table 1:** Inclusion and exclusion criteria

Inclusion criteria	Exclusion criteria
Adults aged 18 years and above	Pediatrics
Both genders	Outside Qassim, Saudi Arabia
From Qassim, Saudi Arabia	Refused to participate in the study
Agreed to participate in the study	

Sample size

The sample size was calculated using the OpenEpi online sample size calculator. It was based on a 95% confidence interval, a 5% margin of error, and the total number of selected people in Qassim, Saudi Arabia. The estimated sample size was 384, and it was adjusted to 422 to compensate for the 10% non-response rate.

Sampling technique

A convenient non-probability sampling technique was employed to collect the data from the participants.

Data collection tools

The study was conducted using an online, self-administered questionnaire via Google Forms. The generated link was shared randomly on social media (e.g., Facebook, WhatsApp, Telegram, and Twitter). The aim of the study was clearly explained at the interface.

A validated questionnaire was used based on previous studies [18,20,21]. The questionnaire contains sociodemographic characteristics of the participants, like age group, sex, nationality, and residence. The questionnaire also includes questions about general public awareness of vitamin D deficiency in Qassim, Saudi Arabia. A standard grading method was used for each variable in this questionnaire: 2 points were given to the correct option, 0 for the incorrect answer, and 1 for neutral.

Pilot study

The questionnaire was pretested in a pilot study over a sample of 20 participants whose results were not included in the study. Some modifications will ensure clarity and easy understanding of the questions.

Ethical considerations

Institutional ethical committee approval was obtained from the Regional Ethics Committee, Qassim, with approval number 607-45-7073. Informed consent was attached to the questionnaire. Once they agree, the questionnaire will appear online. Confidentiality of individual participant information was maintained, and data will not be shared with any public or private organizations.

Statistical analysis

IBM SPSS Statistics for Windows, Version 27.0 (Released 2020; IBM Corp., Armonk, NY, USA) was utilized for both data entry and analysis. Frequency and percentage were obtained to describe categorical data, including sociodemographic and other questions. Regarding knowledge questions, the recording of data was done. Correct answers were assigned a code of 1, while incorrect or neutral answers were assigned a code of 0. A total knowledge score was then calculated for each participant, with a maximum score of 16. Participants scoring 9 or higher were categorized as having a high awareness level of the importance of vitamin D, while those scoring lower were classified as having a poor awareness level. Chi-square and Fisher’s exact tests were employed to assess the association between the knowledge score and sociodemographic data. A statistical significance was observed if the p-value was less than 0.05 or equal to 0.05.

## Results

As shown in Table 2, the majority of participants were women (n = 204, 54.4%), spanning various age groups, with the 21-40 age group representing nearly half of the cohort (n = 185, 49.4%). Additionally, a significant proportion resided in urban areas (n = 293, 78.1%). Concerning marital status, a large portion were either married (n = 150, 40%) or single (n = 141, 37.6%). Moreover, a considerable number held a bachelor’s degree (n = 152, 40.5%) and were employed (n = 156, 41.6%), with a monthly income of less than 10,000 Saudi riyals (n = 177, 47.2%).

**Table 2:** Sociodemographic data (n = 375) in the study population

Variable		n (%)
Gender	Male	171 (45.6)
	Female	204 (54.4)
Age (years)	≤20	61 (16.3)
	21-30	88 (23.5)
	31-40	97 (25.9)
	41-50	76 (20.3)
	51-60	37 (9.9)
	≥61	16 (4.3)
Education	Illiterate	49 (13.1)
	Primary	31 (8.3)
	Intermediate	43 (11.5)
	Secondary	80 (21.3)
	Bachelor’s	152 (40.5)
	Master’s and higher	20 (5.3)
Marital status	Single	141 (37.6)
	Married	150 (40.0)
	Divorced	50 (13.3)
	Widow	34 (9.1)
Residency	Rural	82 (21.9)
	Urban	293 (78.1)
Employment status	Unemployed	100 (26.7)
	Employed	156 (41.6)
	Having own business	48 (12.8)
	Retired	32 (8.5)
	Other	39 (10.4)
Monthly income (SAR)	≤10,000	177 (47.2)
	10,000-14,999	73 (19.5)
	15,000-19,999	62 (16.5)
	20,000-24,999	36 (9.6)
	≥25,000	27 (7.2)

As shown in Table 3, when participants were asked about their personal practices related to vitamin D deficiency, their responses varied. The vast majority had prior knowledge of vitamin D (n = 291, 77.6%) and reported acquaintances with individuals affected by vitamin D deficiency (n = 234, 62.4%). However, fewer respondents answered affirmatively when asked whether they include foods rich in vitamin D in their meals or expose their bodies to sufficient sunlight for vitamin D formation (47.7% and 39.2%, respectively). Despite these poor practices, a significant number admitted to having undergone past checkups for vitamin D (n = 208, 55.5%).

**Table 3:** Personal experiences of participants regarding vitamin D (n = 375)

	Yes, n (%)	No, n (%)	Not sure, n (%)
Have you ever heard of a vitamin D deficiency?	291 (77.6)	51 (13.6)	33 (8.8)
Have you or someone you know been diagnosed with vitamin D deficiency?	234 (62.4)	104 (27.7)	37 (9.9)
Do you eat foods rich in vitamin D?	179 (47.7)	118 (31.5)	78 (20.8)
Do you expose your body to sufficient sunlight?	147 (39.2)	152 (40.5)	76 (20.3)
Have you ever checked your vitamin D levels?	208 (55.5)	117 (31.2)	50 (13.3)
Do you use sunscreen to protect your face from the sun’s rays?	176 (46.9)	147 (39.2)	52 (13.9)
Do you wear hats or other clothing to protect yourself from the sun?	173 (46.1)	146 (38.9)	56 (14.9)

Table 4 revealed that the questionnaire comprised 16 questions designed to examine individuals’ overall knowledge regarding vitamin D deficiency. Participants’ responses varied across different aspects of the topic. A majority correctly acknowledged that vitamin D deficiency is curable (n = 261, 69.6%) and can cause serious health problems if left untreated (n = 242, 64.5%). Additionally, many recognized that vitamin D is formed within the human body (n = 235, 62.7%) and primarily affects bones when deficient (n = 225, 60%). Other facts about vitamin D deficiency also garnered moderately correct response rates, such as its prevalence (n = 217, 57.9%) and vulnerability among older individuals (n = 204, 54.4%). However, fewer participants correctly responded to some questions, including vitamin D in eggs (n = 180, 48%) and its association with areas lacking sunlight or clean air (n = 180, 48% for each). Additionally, a small proportion knew that spending long hours outdoors protects against vitamin D deficiency (n = 164, 43.7%) and that consuming meat is essential for obtaining vitamin D (n = 163, 43.5%). Furthermore, only 41.3% (n = 155) recognized the impact of vitamin D deficiency on immunity, bones, and muscles, while 40.3% (n = 151) understood that sunscreen use could contribute to deficiency. Few knew that vitamin D absorption is not influenced by complexion (n = 134, 35.7%) and is not preserved during pregnancy or lactation (n = 130, 34.7%).

**Table 4:** Responses of participants regarding vitamin D knowledge items

Item	Correct answer	n (%)
Is treatment for vitamin D deficiency possible?	Yes	261 (69.6)
Can vitamin D deficiency lead to serious health issues?	Yes	242 (64.5)
Vitamin D can be acquired within the human body by:	Sunrays	235 (62.7)
Can vitamin D deficiency result in bone disease?	Yes	225 (60.0)
Is vitamin D deficiency a rare problem?	No	217 (57.9)
Older people are more susceptible to vitamin D deficiency.	Yes	204 (54.4)
Seafood is a good source of vitamin D.	Yes	197 (52.5)
Which of the following foods is considered rich in vitamin D?	Eggs	180 (48.0)
People who reside in cloudy climates have an increased risk of vitamin D deficiency.	Yes	180 (48.0)
People who live in areas with air pollution have an increased risk of vitamin D deficiency.	Yes	180 (48.0)
Is vitamin D deficiency more prevalent among individuals who spend extended periods outdoors?	No	164 (43.7)
People who do not consume meat may be more likely to develop a vitamin D deficiency.	Yes	163 (43.5)
Vitamin D deficiency can lead to:	All options	155 (41.3)
Using sunscreen can contribute to vitamin D deficiency.	Yes	151 (40.3)
The risk of vitamin D deficiency may increase in those with fair skin.	No	134 (35.7)
Pregnancy and breastfeeding can protect mothers from vitamin D deficiency.	No	130 (34.7)

The knowledge score of each participant was calculated, indicating that the number of individuals with a good or high awareness score (9 points or more out of 16) was fewer (n = 183, 48.8%) than those with poor or low scores (n = 192, 51.2%). This finding suggests a knowledge gap or areas of misunderstanding among the public regarding vitamin D deficiency as a public health concern.

Assuming a relationship between participants’ sociodemographic characteristics and their awareness level regarding vitamin D deficiency as a public health concern, several variables were found to be associated. Education level emerged as a significant factor, with individuals attaining a university or bachelor’s degree exhibiting the highest knowledge of vitamin D deficiency (n = 90, 59.3%), with a p-value of 0.028. Similarly, place of residence was significantly associated with knowledge level, as individuals residing in cities demonstrated greater awareness of vitamin D deficiency (n = 160, 54.6%) compared to those in villages, with a p-value of 0.001. Employment status also showed an association, with employed individuals displaying higher awareness (n = 88, 56.4%), with a p-value of 0.032. Additionally, monthly income was associated with awareness scores, as participants earning more than 25,000 Saudi riyals per month exhibited higher knowledge scores (n = 16, 59.3%), with a p-value of 0.001. In contrast, gender, age, and marital status did not significantly correlate with individuals’ awareness scores (Table 5).

**Table 5:** The relationship between participants’ sociodemographic data and level of awareness * Pearson’s chi-square test

Variable		Awareness level		p-value^*^
		High (n = 183, 48.8%)	Low (n = 192, 51.2%)	
Gender	Male	84 (49.2)	87 (50.8)	0.918
	Female	99 (48.5)	105 (51.5)	
Age (years)	≤20	28 (46.0)	33 (54.0)	0.276
	21-30	50 (56.8)	38 (43.2)	
	31-40	48 (49.5)	49 (50.5)	
	41-50	29 (38.2)	47 (61.8)	
	51-60	20 (54.1)	17 (45.9)	
	≥61	8 (50.0)	8 (50.0)	
Education	Illiterate	21 9 (42.9)	28 (57.1)	0.028
	Primary	12 (38.7)	19 (61.3)	
	Intermediate	15 (34.9)	28 (65.1)	
	Secondary	36 (45.0)	44 (55.0)	
	Bachelor’s	90 (59.3)	62 (40.7)	
	Other	9 (45.0)	11 (55.0)	
Marital status	Single	73 (51.8)	68 (48.2)	0.085
	Married	79 (52.7)	71 (47.3)	
	Divorced	20 (40.0)	30 (60.0)	
	Widow	11 (32.4)	23 (67.6)	
Residency	Rural	23 (28.1)	59 (71.9)	0.001
	Urban	160 (54.6)	133 (45.4)	
Employment status	Unemployed	49 (49.0)	51 (51.0)	0.032
	Employed	88 (56.4)	68 (43.6)	
	Having own business	20 (41.7)	28 (58.3)	
	Retired	9 (28.1)	23 (71.9)	
	Other	17 (43.6)	22 (56.4)	
Monthly income (SAR)	≤10,000	104 (58.8)	73 (41.2)	0.001
	10,000-14,999	30 (41.1)	43 (58.9)	
	15,000-19,999	21 (33.9)	41 (66.1)	
	20,000-24,999	12 (33.4)	24 (66.6)	
	≥25,000	16 (59.3)	11 (40.7)	

**Figure 1:**
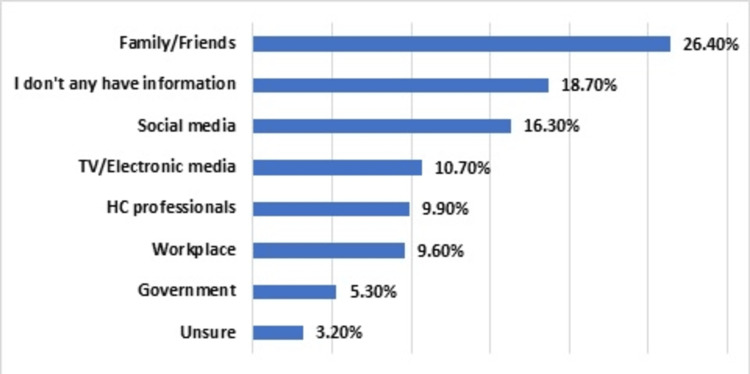
Source of information about vitamin D deficiency (n = 375)

Participants’ sources of information about vitamin D deficiency varied. Families and/or friends primarily constituted the main source of knowledge (n = 99, 26.4%). Other sources, such as social media (n = 61, 16.3%), TV/electronic media (n = 40, 10.7%), health staff (n = 37, 9.9%), and governmental entities (n = 20, 5.3%) were also frequently cited. However, a notable portion reported having no information about the topic (n = 70, 18.7%), while fewer individuals (n = 12, 3.2%) were unsure about their responses (Figure 1).

**Figure 2:**
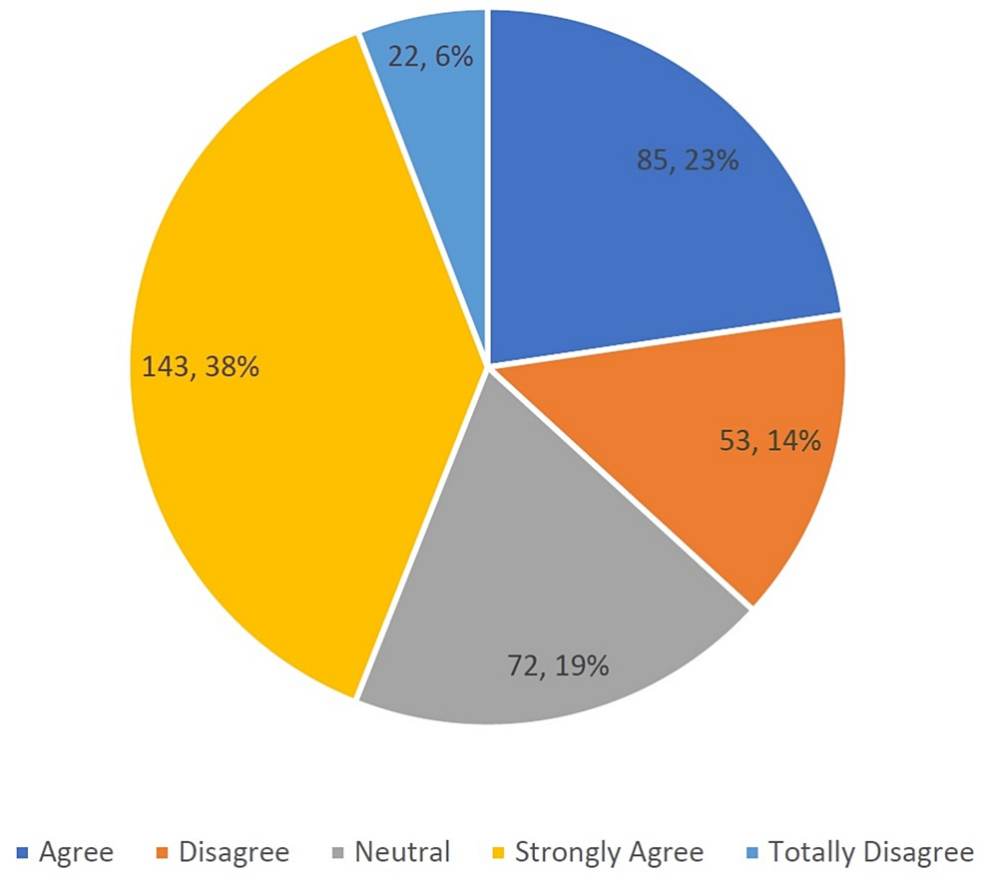
Participants' self-reported level of knowledge of vitamin D deficiency (n = 375)

Participants were asked about their perceived level of knowledge regarding vitamin D deficiency. More than half indicated they possessed considerable knowledge on this topic (n = 143), 38.1% strongly agreed (n = 85), and 22.7% agreed. Fewer respondents admitted to lacking a good understanding of this health issue (n = 53), 14.1% disagreed (n = 22), and 5.9% strongly disagreed. However, a notable portion had neutral opinions (n = 72, 19.2%) (Figure 2).

## Discussion

Vitamin D, which consists of D2 and D3, is vital for various bodily functions and homeostasis [22]. Its deficiency is a significant public health concern worldwide, impacting various aspects of health and well-being. Our study aimed to assess the public awareness of vitamin D deficiency in Qassim, Saudi Arabia, examining its relationship with sociodemographic factors. We found that there were gaps in the knowledge of the population about vitamin D deficiency. Less than half (47% of vitamin D-rich foods) are in their diet. Similarly, a little less than half (48.8%) had adequate knowledge of vitamin D deficiency. Education, place of residence, employment status, and income levels were significantly associated with knowledge.

Notably, our results revealed mixed awareness and practices among the participants. A majority (77.6%) reported prior knowledge of vitamin D and acquaintance with affected individuals (62.4%). These findings show that the majority of our participants are aware of vitamin D. However, a previous study by Alemu and Varnam revealed that 28% of them (n = 61) had never heard about vitamin D [23]. Fewer people engage in behaviors that promote vitamin D sufficiency, such as consuming foods rich in vitamin D (47.7%) or exposure (39.2%) to sunlight. Similarly, Tariq et al. show that over 40% spent ≥1 hour outdoors on workdays, while around 17% spent ≤15 minutes outdoors, with an overall 57% practicing sunbathing to gain enough vitamin D [24]. Despite these shortcomings, a significant number (55.5%) had undergone checkups for vitamin D, indicating a proactive approach to managing their health. Similarly, Hamhoum and Aljefree show that 99.6% of participants are willing to undergo a test for vitamin D if a medical condition demands it, showing that they are concerned about their health and its management [25].

Participants primarily relied on informal sources, such as family and friends, for information about vitamin D deficiency, followed by social media and electronic media. This underscores the importance of leveraging these channels for disseminating accurate information and promoting healthy behaviors. However, a study by Kambal et al. shows that the source of information about vitamin D knowledge is a healthcare provider (32.2%) among participants [26]. A notable proportion (18.7%) reported having no information about the topic, highlighting the need for targeted educational campaigns to reach underserved populations. Similarly, Alshamsan and Bin-Abbas revealed that most vitamin D-deficient cases were unaware of vitamin D sources [27].

Notably, most participants (69.6%) in our study correctly recognized vitamin D deficiency as treatable. They linked it to serious health issues (64.5%) like osteoporosis, demonstrating awareness of its role in bone health and broader medical conditions. Similarly, Holick shows that vitamin D deficiency can lead to osteomalacia and rickets in children and osteomalacia in adults [28]. Moreover, Lee et al. show that vitamin D deficiency seems to predispose to hypertension, diabetes, metabolic syndrome, left ventricular hypertrophy, congestive heart failure, and chronic vascular inflammation [29].

Understanding that vitamin D is synthesized and acquired internally (62.7%) and crucial for bone health (60%) reflects basic knowledge of its physiology and role in skeletal health, supported by existing literature. DeLuca shows that vitamin D stimulates calcium and phosphorus absorption in the intestines, bone calcium mobilization, and renal calcium reabsorption, mainly regulated by the parathyroid hormone [30].

However, there are notable gaps in knowledge among the participants. For instance, fewer individuals correctly responded to questions regarding sources of vitamin D, such as its presence in eggs (48%) and its association with sunlight exposure (48%) and clean air (48%). This highlights a need for education on dietary sources of vitamin D and the factors influencing its synthesis in the body, such as sun exposure. Huang et al. show that dietary vitamin D is crucial to addressing deficiency in tropical regions, while sunlight exposure is key in subtropical areas. Promoting both strategies is essential for healthcare programs [31].

Furthermore, a minority (41.3%) of participants acknowledged vitamin D deficiency’s broader health effects beyond bones, indicating a need for awareness. Emerging research links vitamin D to immune function and chronic diseases, highlighting its significance. Similarly, Sîrbe et al. show calcitriol’s immunologic effects and link low serum 25(OH)D levels to an increased risk of immune-related diseases [32].

Similarly, few participants (40.3%) recognized sunscreen’s impact on vitamin D synthesis, highlighting the need to educate about balancing sun protection and vitamin D production. Raymond-Lezman and Riskin show that sunscreen, contrary to belief, does not significantly inhibit vitamin D status as it reduces UV exposure and subsequently lowers vitamin D production [33].

Moreover, our study identified several sociodemographic factors associated with awareness levels regarding vitamin D deficiency. Education level emerged as a significant predictor, with individuals with bachelor’s degrees demonstrating higher awareness. This finding aligns with previous research indicating that higher education is linked to better health literacy and awareness of preventive health measures. Similarly, Zaremba and Conduit-Turner show that university degrees are more likely to have knowledge scores about vitamin D awareness [34].

Similarly, urban residence, employment status, and higher income were associated with greater awareness, suggesting that access to resources and exposure to health information contribute to heightened awareness. Other individual features can affect a person’s level of awareness regarding vitamin D deficiency. Al-Daghri et al. revealed that physically active individuals showed higher vitamin D awareness [35]. Alibrahim et al. (2024) show age and occupation were significantly correlated with adequate awareness of vitamin D [36].

There are some strengths to our study. We recruited participants from the general population, unlike previous studies from Saudi Arabia, where recruitment was done in health facilities. We used a comprehensive questionnaire to assess the knowledge of the population. However, there are certain limitations that need consideration while interpreting the results of this study. Firstly, the cross-sectional design limits the ability to establish causality or temporal relationships between variables. Secondly, the reliance on self-reported data may introduce biases such as social desirability bias or recall bias. Another limitation of our study is online recruitment, which could have excluded individuals who are not active on social media and thus affected the generalizability of our findings. Future studies must be conducted on a large scale and focus on interventional studies and their effectiveness in improving population awareness about vitamin D deficiency.

## Conclusions

Our study provides valuable insights into the general public’s awareness of vitamin D deficiency in Qassim, Saudi Arabia, and highlights the need for targeted interventions to address knowledge gaps and promote healthy behaviors. Our study suggests targeted educational campaigns to address misconceptions about vitamin D deficiency, improved screening and supplementation access, and policy initiatives promoting outdoor activity and food fortification in Qassim, Saudi Arabia. These initiatives require collaborative efforts across sectors. Future research could utilize longitudinal designs and objective measures of awareness and practices to overcome these limitations.
